# Temporal Changes in Key Hematological and Inflammatory‐Immune Markers Among Elderly Patients Following Hip Fracture: A Five‐Day Serial Assessment

**DOI:** 10.1111/os.70288

**Published:** 2026-03-19

**Authors:** Yuening Han, Shanshan Zhang, Xinqun Cheng, Yuqing Li, Chengsi Li, Yingze Zhang, Yanbin Zhu, Cici Bai, Xiuting Li

**Affiliations:** ^1^ Economic Development Institute of Nankai University Nankai University Tianjin P. R. China; ^2^ Department of Orthopaedic Surgery Hebei Medical University Third Hospital Shijiazhuang Hebei P. R. China; ^3^ Hebei Orthopaedic Research Institute Key Laboratory of Biomechanics of Hebei Province, Hebei Medical University Third Hospital Shijiazhuang Hebei P. R. China

**Keywords:** elderly patients, hematological markers, hip fracture, perioperative management, prognostic stratification, temporal trends

## Abstract

**Objective:**

Hip fracture is a severe injury in the elderly population and can trigger a strong physiologic stress reaction with potential impact on clinical outcomes. However, few data are available on the temporal evolution of hematologic parameters after this injury. This study aimed to evaluate the temporal trends of key hematological and inflammatory‐immune markers in elderly patients with hip fractures.

**Methods:**

A retrospective cohort study was conducted among elderly patients with hip fractures managed at a tertiary referral center from January 2022 to October 2024. Included patients were required to have both complete serial hematological measurements obtained during the first 5 days post‐fracture and relevant clinical data. We used generalized estimating equation models for repeated measurements to describe the temporal trends of key hematological markers, with analyses stratified by fracture type and age group.

**Results:**

A total of sixty patients were included, with a mean age of 80 ± 7.4 years (range: 65–96 years) and 68.3% females (*n* = 41). Within the first 1–5 days post‐fracture, hemoglobin decreased by a mean of 9.66 g/L, hematocrit by 3.10 percentage points, neutrophil percentage by 8.12 percentage points, neutrophil count by a mean of 2.41 × 10^9^/L, and neutrophil‐to‐lymphocyte ratio (NLR) by 3.07 (all *p*‐values < 0.001). Conversely, lymphocyte and monocyte counts exhibited a biphasic change, peaking on day 4 prior to subsequent decline. Subgroup analyses revealed that monocyte levels demonstrated significant interactions between time and fracture type (*p* = 0.036), whereas both lymphocytes (*p* = 0.034) and monocytes (*p* = 0.012) exhibited significant interactions between age and time.

**Conclusions:**

Hemoglobin, hematocrit, neutrophil percentage, neutrophil count, and NLR progressively decrease during days 1–5 after hip fracture in older patients, whereas lymphocyte and monocyte counts exhibit biphasic patterns and vary significantly according to fracture type and age. These findings may help clinicians in interpreting early post‐fracture laboratory dynamics and provide a basis for future outcome‐oriented validation.

## Graphical abstract

This study found that key hematological parameters showed temporal patterns: hemoglobin, hematocrit, neutrophil percentage, neutrophils, and NLR significantly decreased from days 1 to 5 (all *p* < 0.001), while lymphocytes and monocytes exhibited biphasic dynamics. Monocytes had a significant interaction with time and fracture type, while lymphocytes and monocytes had a significant interaction with both age and time.

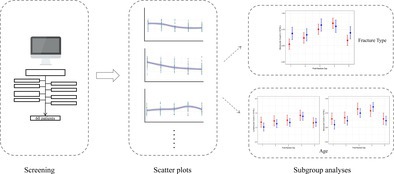

## 1 Introduction

Hip fracture is a common and serious injury in older adults, resulting in high mortality and morbidity, and a substantial burden on patients and health systems [1, 2]. Beyond the fracture itself, the associated intense pain and physiological stress response may exacerbate tissue damage and precipitate systemic decompensation in this vulnerable population. Furthermore, fracture repair surgery during this unstable period may amplify physiological stress (“second hit”), potentially resulting in a substantially increased risk of adverse outcomes [3].

Recent evidence suggests that the serial changes in routinely available hematological and inflammatory‐immune markers after hip fracture are an important reflection of early post‐fracture physiological perturbations [3, 4], potentially providing a practical window into patients' evolving status [5]. While these markers reflect systemic conditions, their changing patterns following hip fracture profoundly reveal the unique pathophysiological process of this injury. For example, the progressive alterations in hematological markers due to ongoing post‐fracture blood loss are linked to preoperative anemia risk and hemorrhage volume, whereas the trauma‐activated immunoinflammatory network integrates information from both the innate and adaptive immune systems, quantifying the systemic inflammatory burden [6, 7]. Accordingly, serial monitoring of these markers, especially their short‐term trajectories post‐fracture, may therefore complement perioperative assessments and optimization. However, most previous studies have relied on single time‐point measurements and have reported inconsistent associations with clinical outcomes [8, 9]. In international guidelines, surgery is generally recommended within 24–48 h of admission [10], while evidence regarding the impact of surgical delay on outcomes remains mixed [11, 12]. A clearer description of early preoperative laboratory trajectories may help contextualize routine test results and provide descriptive reference information for future outcome‐driven research.

Therefore, the aims of this study were (i) to systematically describe the temporal trajectories of key hematological and inflammatory‐immune markers during the first 5 days after fracture in elderly patients with hip fracture, and (ii) to determine whether these trajectories differed by fracture type and age group.

## 2 Materials and Methods

### 2.1 Study Population

Elderly patients aged 65 years or above with low‐energy hip fracture who were hospitalized in the tertiary referral center between January 2022 and October 2024 were retrospectively reviewed via the electronic medical record system. Inclusion criteria were the follows: (1) Hospital admission on the day of fracture occurrence; (2) no surgical intervention or blood transfusion within the first five days post‐fracture and (3), availability of complete serial laboratory results and clinical data. Exclusion criteria were: (1) Pathological fracture (e.g., due to neoplasia) or old/chronic fractures; (2) active malignancy at the time of admission; (3) polytrauma or concomitant fractures at other anatomical sites; (4) receipt of any blood transfusion; (5) pre‐existing hematological disorders or diagnosed immune system diseases; (6) systemic infectious diseases on admission; (7) severe dysfunction of major organs (e.g., advanced heart, liver, or renal failure); or (8) incomplete medical or laboratory records.

This study was conducted in accordance with the Helsinki Declaration and received ethical approval from the local institutional review board (W2024‐014‐1). With all data fully anonymized in this retrospective study, a waiver of informed consent was obtained.

### 2.2 General Information

In this study, we collected all the study variables from the electronic medical record system, including demographics, injury‐related data, and laboratory test results. In particular, the extracted data were gender, age, fracture type (femoral neck or intertrochanteric), Age‐adjusted Charlson Comorbidity Index (ACCI), injured side, time from injury to hospital admission, date of injury and key hematological parameters, including hemoglobin (Hb) and hematocrit (Hct), neutrophil percentage (NEU%), neutrophil count (neutrophils), lymphocyte count (lymphocytes), monocyte count (monocytes), and neutrophil‐to‐lymphocyte ratio (NLR).

This retrospective study extracted and defined all clinical variables based on records from the electronic medical record system. The definitions of hypertension, diabetes, anemia, pneumonia, and osteoporosis were based on previously recorded diagnoses in the medical history. Cardiovascular diseases included conditions such as heart failure, myocardial infarction, and coronary artery disease. Cerebrovascular diseases covered ischemic stroke, hemorrhagic stroke, and transient ischemic attack, among other related conditions. Liver diseases, kidney diseases, and gallbladder diseases were defined according to relevant specialist diagnoses recorded in the system. Thrombosis included deep vein thrombosis and pulmonary embolism.

### 2.3 Calculation of ACCI

According to previous literature [13], Charlson Comorbidity Index (CCI) was calculated by counting each comorbidity (myocardial infarction, congestive heart failure, cerebrovascular disease, peripheral vascular disease, dementia, rheumatoid disease, peptic ulcer disease, diabetes (with/without complications), chronic pulmonary disease, liver disease (mild/moderate and severe), hemiplegia, moderate and severe renal disease, solid tumor (with/without metastasis), leukemia, lymphoma and acquired immunodeficiency syndrome (AIDS)) according to its weighted score. Then, ACCI was calculated on this basis by adding 1 point for every 10 years of age for patients over 40 years (0 point for ≤ 40 years, 1 point for 41–50 years, 2 points for 51–60 years, 3 points for 61–70 years, and 4 points for > 70 years) [14].

### 2.4 Selection of Hematological Markers

The selection of hematological markers in this study was based on the following considerations. First, they collectively reflect the multifaceted physiological stress response after trauma, including anemia caused by blood loss and systemic immune‐inflammatory reactions; second, all the markers are routine clinical tests, possessing the advantages of ready availability and low cost, facilitating real‐world dynamic clinical monitoring. Third, they allow for the establishment of a basic temporal reference framework for future inclusion of more specific biomarkers.

### 2.5 Definition of Post‐Fracture Time Points and Blood Sampling Schedule

Taking the specific date of the fracture occurrence as the reference point (D0). “Day 1 after fracture (D1)” is defined as the first calendar day after the fracture occurred, “Day 2 after fracture (D2)” to “Day 5 after fracture (D5)” follow the same pattern. Additionally, since this study is retrospective, the blood samples were drawn on the day they were tested, at a time determined by the clinical team based on the daily work schedule and the patient's condition. It was not strictly fixed at the same time every day. This design aims to capture the early physiological dynamic changes trend after the fracture, measured in “days,” and reflect the monitoring mode in the real clinical environment.

### 2.6 Statistical Methods

The Kolmogorov–Smirnov test was applied to assess the normality of continuous variables. Continuous variables, when in normal distribution, were presented as means ± SD and analyzed using the Student's *t*‐test; otherwise, they were presented as median and interquartile range and analyzed using the Mann–Whitney test. Categorical covariables were expressed as numbers (%) and analyzed using the χ2‐test. We employed multiple imputation to handle missing data and a generalized estimating equations (GEE) model to analyze temporal trends. Specifically, this GEE model used a Gaussian distribution with an identity link and an exchangeable correlation structure. We employed robust standard errors for all statistical inferences. For visualization, “day” was treated as continuous when plotting fitted curves. No covariates were adjusted for, consistent with the descriptive aim of estimating the unadjusted, overall change trajectory of each marker. To control for multiple testing across the four adjacent day pairs, a Bonferroni correction was applied. Additionally, the study compared various marker levels among patients stratified by fracture type (femoral neck vs. intertrochanteric) and by age group (< 80 vs. ≥ 80 years).

Statistical analysis was performed using IBM SPSS Statistics version 21.0 (IBM, Armonk, New York, USA) and R software version 4.4.2 (R Foundation for Statistical Computing, Vienna, Austria). A two‐sided 5% significance level was prespecified and pairwise comparisons were adjusted for multiple comparisons using a two‐sided 1.25% significance level.

## 3 Results

### 3.1 General Characteristics of Patients

**FIGURE 1:**
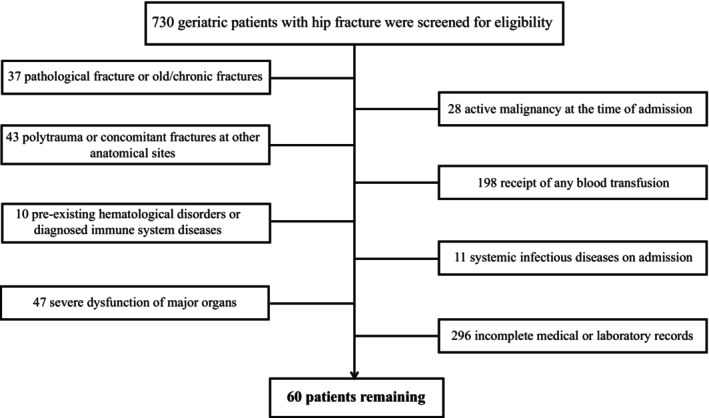
The patient flow chart in our study.

Between January 2022 and October 2024, a total of 730 elderly patients with hip fractures were screened for eligibility. Following application of the inclusion and exclusion criteria, 60 patients were ultimately enrolled in the final analysis (Figure 1). Exclusion criteria comprised the following: pathological fractures or old/chronic fractures (*n* = 37), active malignancy at the time of admission (*n* = 28), polytrauma or concomitant fractures at other anatomical sites (*n* = 43), receipt of blood transfusion (*n* = 198), pre‐existing hematological disorders or diagnosed immune system diseases (*n* = 10), systemic infectious diseases on admission (*n* = 11), severe dysfunction of major organs (*n* = 47), and incomplete medical or laboratory records (*n* = 296).

The mean age of the included patients was 80.0 ± 7.4 years (range: 65–96 years), with 19 males (31.7%) and 41 females (68.3%). Left‐sided injuries accounted for 36 cases (60%), while 24 cases (40%) involved the right side. The average interval from injury to hospital admission was 6 h (Table 1).

**TABLE 1:** Baseline characteristics of patients with hip fracture.

Variable	Total patients (*n* = 60)
Age (years)	80.0 ± 7.4
Gender, *n*	
Male	19 (31.7%)
Female	41 (68.3%)
Fracture type, *n*	
Femoral Neck Fracture	35 (58.3%)
Intertrochanteric Fracture	25 (41.7%)
ACCI, *n*	
4–5	22 (36.7%)
≥ 6	38 (63.3%)
Injured side, *n*	
Left	36 (60%)
Right	24 (40%)
Time from injury to admission (h)	6.0 (5.8)
Hypertension	41 (68.3%)
Diabetes	22 (36.7%)
Cerebrovascular disease	42 (70.0%)
Heart disease	44 (73.3%)
Pneumonia	20 (33.3%)
Liver disease	9 (15%)
Kidney disease	7 (11.7%)
Gallbladder diseases	7 (11.7%)
Osteoporosis	33 (55%)
Thrombus	25 (41.7%)
Anemia	29 (48.3%)

### 3.2 Overall Trends of Multiple Hematological Markers

**FIGURE 2:**
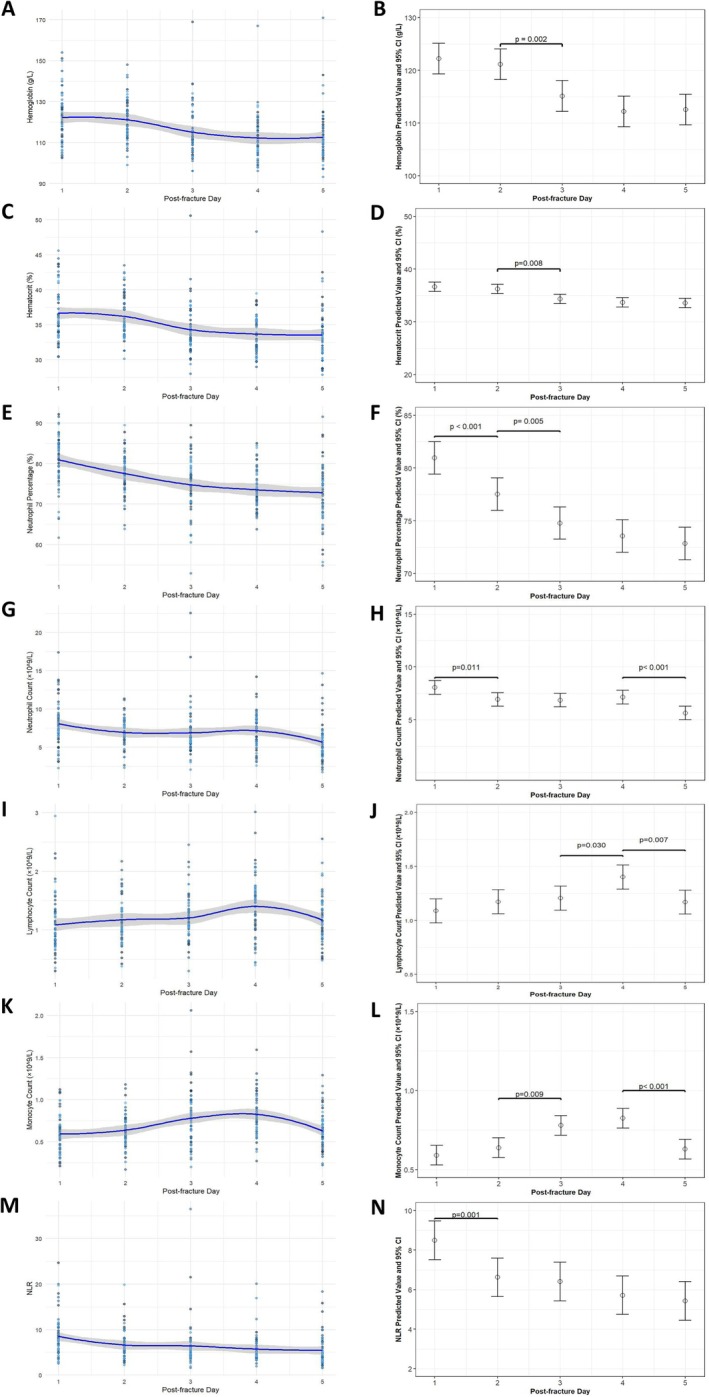
Scatter plots of the first 1–5 days post‐fracture for each hematological marker with locally weighted scatter plot smoothing best‐fit curve and average values with 95% confidence intervals.

Hemoglobin and hematocrit decreased significantly over time (both *p* < 0.001), with maximal declines of 9.66 g/L and 3.1% between peak and nadir values, respectively (Table 2). Among them, both hemoglobin and hematocrit showed significant decreases from D2 to D3 (Δ = −6.03 g/L, *p* = 0.002; Δ = −1.9%, *p* = 0.008) (Figure 2A–D). This progressive decline underscores the importance of serial hematological monitoring after fracture to guide decisions regarding potential transfusion or investigation for occult bleeding.

**TABLE 2:** Changes in each hematological marker in the first 1–5 days after fracture.

Hematological markers	Post‐fracture day	Predicted value	95% confidence interval
Hemoglobin (g/L)	Day1	122.22	119.29, 125.14
	Day2	121.15	118.22, 124.07
	Day3	115.12	112.20, 118.05
	Day4	112.20	109.27, 115.12
	Day5	112.56	109.64, 115.49
Hematocrit (%)	Day1	36.66	35.78, 37.54
	Day2	36.22	35.34, 37.10
	Day3	34.32	33.44, 35.20
	Day4	33.68	32.81, 34.56
	Day5	33.56	32.68, 34.44
Neutrophils percentage (%)	Day1	80.95	79.41, 82.49
	Day2	77.53	75.99, 79.07
	Day3	74.78	73.24, 76.32
	Day4	73.55	72.01, 75.09
	Day5	72.83	71.29, 74.37
Neutrophil Count (×10^9/L)	Day1	8.05	7.40, 8.70
	Day2	6.92	6.28, 7.57
	Day3	6.85	6.21, 7.50
	Day4	7.15	6.50, 7.79
	Day5	5.64	5.00, 6.29
Lymphocyte Count (×10^9/L)	Day1	1.09	0.98, 1.20
	Day2	1.17	1.06, 1.28
	Day3	1.21	1.09, 1.32
	Day4	1.40	1.29, 1.51
	Day5	1.17	1.06, 1.28
Monocyte Count (×10^9/L)	Day1	0.59	0.53, 0.65
	Day2	0.64	0.58, 0.70
	Day3	0.78	0.72, 0.84
	Day4	0.83	0.76, 0.89
	Day5	0.63	0.57, 0.69
Neutrophil‐to‐lymphocyte ratio	Day1	8.50	7.52, 9.48
	Day2	6.62	5.65, 7.60
	Day3	6.41	5.43, 7.39
	Day4	5.72	4.74, 6.70
	Day5	5.43	4.45, 6.41

The neutrophil percentage and neutrophils both showed a gradually decreasing trend (both *p* < 0.001). Neutrophil percentage peaked on D1, declined to normal levels by D3, and reached its nadir on D5 (Δ = −8.12%) (Table 2). Significant reductions occurred on D1–D2 (Δ = −3.42%, *p* < 0.001) and D2–D3 (Δ = −2.75%, *p* = 0.005), with stabilization thereafter (Figure 2E–F). Neutrophils remained elevated until normalization on D5. Significant decreases were observed on D1‐D2 (Δ = −1.13 × 10^9^/L, *p* = 0.011) and D4‐D5 (Δ = −1.51 × 10^9^/L, *p* < 0.001), while counts plateaued during D2‐D4 (*p* = 0.985 for both) (Figure 2G–H). Although showing a downward trend, the neutrophil percentage and count in the early post‐fracture period remained elevated. This indicates that a significant systemic inflammatory response persisted during the early window period after the fracture. This finding suggests the importance of conducting a clinical assessment at this stage, as the persistent inflammatory state may be related to the patient's clinical progression.

Lymphocytes and monocytes exhibited a biphasic change (both *p* < 0.001). Specifically, its characteristic is that it begins to rise post‐fracture and reaches its peak on D4, then starts to decline. Lymphocytes returned to normal levels after D1. From D3 to D4, lymphocytes showed a significant increase (Δ = +0.19 × 10^9^/L, *p* = 0.030), followed by a significant decrease from D4 to D5 (Δ = −0.23 × 10^9^/L, *p* = 0.007). Monocytes exhibited different patterns: their numbers remained elevated above normal levels after D1. During D2‐D3, monocytes rose significantly (Δ = +0.14 × 10^9^/L, *p* = 0.009), but then dropped significantly from D4‐D5 (Δ = −0.20 × 10^9^/L, *p* < 0.001) (Figure 2I–L). This biphasic pattern reflects a dynamic immune response to trauma, indicating a period of potential immunomodulation that could be relevant for perioperative immune function and infection susceptibility.

NLR showed a significant trend of decreasing over time (*p* < 0.001), with the value decreasing from peak to trough by 3.07 (Table 2). Among them, NLR showed significant decreases from D1 to D2 (Δ = −1.88, *p* = 0.001), and the changes on other days were not significant. (Figure 2M,N). Although the NLR is showing a downward trend, its value remains persistently high. This trend indicates that the patient is in a continuous state of systemic inflammation and stress. The dynamic changes of NLR can provide a quantitative reference for clinical assessment of the severity of the patient's physiological stress.

### 3.3 Subgroup Analysis

This study further investigated the interaction between these markers across different fracture types and age stratification scenarios. Tables 3 and 4 details the baseline characteristics of hip fracture patients classified by fracture type and age.

**TABLE 3:** Baseline characteristics of patients with hip fracture classified by fracture type.

Variable	Femoral neck fracture group (*n* = 35)	Intertrochanteric Fracture Group (*n* = 25)	*p*
Age (years)	81.7 ± 7.2	77.8 ± 7.3	0.459
Gender, *n*			0.963
Male	11 (31.4%)	8 (32.0%)	
Female	24 (68.6%)	17 (68.0%)	
ACCI, *n*			0.124
4–5	10 (28.6%)	12 (48.0%)	
≥ 6	25 (71.4%)	13 (52.0%)	
Injured side, *n*			0.109
Left	18 (51.4%)	18 (72.0%)	
Right	17 (48.6%)	7 (28.0%)	
Time from injury to admission (h)	7.0 (6.0)	6.0 (5.0)	0.346
Hypertension	22 (62.9%)	19 (76.0%)	0.284
Diabetes	12 (34.3%)	10 (40.0%)	0.651
Cerebrovascular disease	24 (68.6%)	18 (72.0%)	0.775
Heart disease	25 (71.4%)	19 (76.0%)	0.693
Pneumonia	11 (31.4%)	9 (36.0%)	0.711
Liver disease	5 (14.3%)	4 (16.0%)	0.855
Kidney disease	5 (14.3%)	2 (8.0%)	0.460
Gallbladder diseases	5 (14.3%)	2 (8.0%)	0.460
Osteoporosis	17 (48.6%)	16 (64.0%)	0.239
Thrombus	11 (31.4%)	14 (56.0%)	0.060
Anemia	14 (40%)	15 (60.0%)	0.129

**TABLE 4:** Baseline characteristics of patients with hip fracture classified by age.

Variable	Age(years)		*p*
	< 80 (*n* = 26)	≥ 80 (*n* = 34)	
Age (years)	72.9 ± 3.8	85.5 ± 4.1	< 0.001
Gender, *n*			0.322
Male	10 (38.5%)	9 (26.5%)	
Female	16 (61.5%)	25 (73.5%)	
ACCI, *n*			0.428
4–5	11 (42.3%)	11 (32.4%)	
≥ 6	15 (57.7%)	23 (67.6%)	
Injured side, *n*			0.202
Left	18 (69.2%)	18 (53.0%)	
Right	8 (30.8%)	16 (37.1%)	
Time from injury to admission (h)	6.5 (5.3)	6.0 (5.5)	0.290
Hypertension	18 (69.2%)	23 (67.6%)	0.896
Diabetes	10 (38.5%)	12 (35.3%)	0.801
Cerebrovascular disease	22 (84.7%)	20 (58.8%)	0.037
Heart disease	17 (65.4%)	27 (79.4%)	0.227
Pneumonia	7 (26.9%)	13 (38.2%)	0.359
Liver disease	7 (26.9%)	2 (5.9%)	0.037
Kidney disease	3 (11.5%)	4 (11.8%)	0.978
Gallbladder diseases	2 (7.7%)	5 (14.7%)	0.409
Osteoporosis	14 (53.8%)	19 (55.9%)	0.875
Thrombus	9 (34.6%)	16 (47.1%)	0.334
Anemia	10 (38.5%)	19 (55.9%)	0.183

**FIGURE 3:**
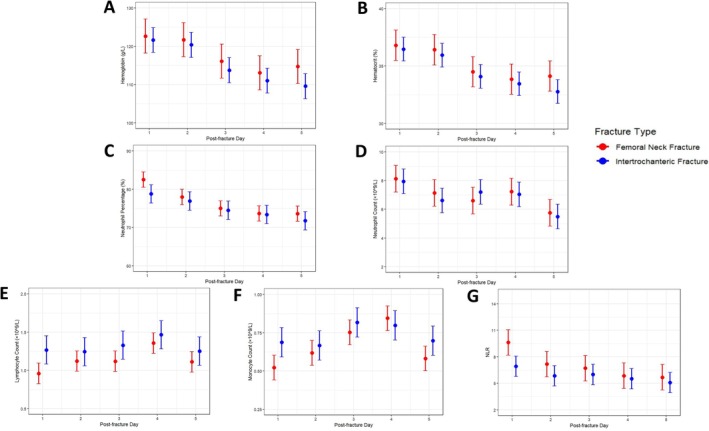
The changes in each hematological marker during the first 1–5 days post‐fracture are shown graphically divided by fracture type.

Further analysis showed that monocytes (*p* = 0.036) exhibited significant interactions over time and fracture type (Figure 3F). Both groups of hip fracture patients exhibited biphasic changes in monocytes, but their peak times differed. Specifically, the monocytes in the intertrochanteric fracture group peaked on D3, while the other group showed a different pattern with their value reaching maximum levels on D4. Monocyte counts were consistently higher in the intertrochanteric fracture group, but the femoral neck group exhibited higher counts on D4.

**FIGURE 4:**
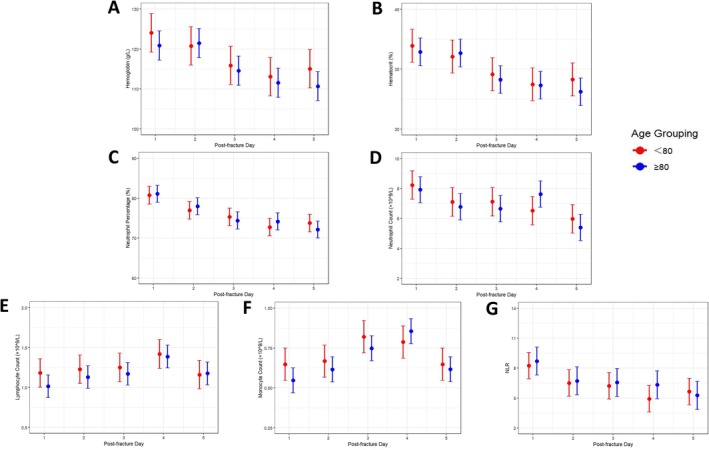
The changes in each hematological marker during the first 1–5 days post‐fracture are shown graphically divided by age.

The analysis further revealed that lymphocyte (*p* = 0.034) and monocyte (*p* = 0.012) counts exhibited significant interactions with age and time (Figure 4E,F). Both age groups shared a lymphocyte peak on D4, with the level in the ≥ 80 age group remaining consistently lower than that in the < 80 age group until D5 when they became comparable. The monocyte peak, however, differed by age, occurring on D3 in the < 80 age group and D4 in the ≥ 80 age group. Monocytes were consistently higher in the < 80 age group compared to the ≥ 80 group across all observation days except for D4.

## 4 Discussion

### 4.1 Main Findings

This study revealed dynamic changes in key hematological markers during the first five days after hip fracture in elderly patients. We observed a steady decline in hemoglobin and hematocrit over time. Meanwhile, neutrophil counts, neutrophil percentage, and the NLR remained elevated despite a gradual decrease. Lymphocyte and monocyte counts showed a biphasic trajectory, peaking around D4 prior to decline.

### 4.2 Underlying Mechanisms

Several physiological mechanisms may explain the observed temporal patterns. First, the early decline in hemoglobin and hematocrit levels is likely multifactorial. Occult hidden blood loss into the fracture site and surrounding soft tissues can accumulate during the first few days after injury [15]. For example, Smith et al. [16] showed that up to 400 mL of hidden blood loss due to initial trauma can occur on day 3 after fracture. Blood dilution from intravenous fluid administration during resuscitation may also unmask or accentuate the decline in hemoglobin and hematocrit [17]. In addition, the release of inflammatory cytokines after injury may suppress erythropoiesis and alter iron handling, further contributing to early anemia [18]. Second, changes in neutrophils and the NLR may reflect the acute stress and inflammatory response after injury. Sympathetic activation and cytokine signaling can promote neutrophils demargination, bone marrow release, and recruitment to injured tissues [19]. In hip fracture setting, the vulnerable elderly patients with multimorbidity or subclinical infection can further amplify neutrophils predominance through pathogen‐associated molecular pattern [20]. Third, a transient elevation of monocytes may indicate early immune activation followed by redistribution or depletion [21]. This temporal difference between monocyte and lymphocyte trajectories may reflect variations in baseline frailty, fracture stability, post‐fracture bleeding burden, and age‐related immune responsiveness [22, 23]. In very old patients, immunosenescence may contribute to a blunted or delayed recovery of immune cell subsets [21]. In addition, age‐related thymic involution may reduce naïve T‐cell output and adaptive immune reserve, which could further influence the observed lymphocyte‐related changes [24, 25].

### 4.3 Comparison With Other Studies

Our findings are consistent with previous literature reporting on changes in hematological markers after trauma. For example, similar neutrophil and monocyte patterns have been described after fractures and surgery [26]. A single‐cell transcriptomic study focused on elderly hip fracture patients also showed a gradual decrease in the proportion of neutrophils during the perioperative period [4]. However, another study showed that lymphocytes in most trauma patients showed a significant increase within 3 h after injury, but a significant decrease over the 6–72 h, which contradicts our findings [27]. This phenomenon may be attributed to differences in trauma type, severity, patient age and clinical interventions. Severe trauma triggers systemic inflammation, which leads to a large increase in lymphocytes followed by a rapid decrease. And for older patients, a combination of reduced immunity, delayed response and conservative treatment also contributes to this phenomenon. A prospective study has shown that lymphocyte counts decrease after trauma in severely injured patients, but may normalize within 48 h [28], however, whether this pattern applies to elderly hip fracture patients is unclear. In our data, lymphocyte and monocyte dynamics exhibited distinct temporal trajectories that varied by fracture type and age group, underscoring the need for further investigation.

International guidelines explicitly recommend that hip fracture patients should be operated within 24–48 h [10], but perioperative decision‐making in this vulnerable population must balance the benefits of early surgery with the need for physiological optimization. It should be noted that, although our cohort consisted of those who had complete serial laboratory measurements within the first 5 days post‐fracture before surgery, it was not designed to infer causality regarding surgical timing and did not compare early versus delayed operative strategies. Therefore, these descriptive data are intended to complement guideline‐based pathways and should not be interpreted as evidence supporting the delayed surgery.

The trajectories of key markers observed in this study may help contextualize routine laboratory results and can be framed as a cautious, hypothesis‐generating “trend‐risk‐action” heuristic for perioperative assessment. Specifically, a steeper‐than‐expected decline in hemoglobin/hematocrit over consecutive days often suggests ongoing hidden blood loss or hemodilution. In clinical practice, continuous monitoring, assessment of volume status, and timely planning of anemia management should be implemented to optimize preoperative preparation. Persistently elevated neutrophil‐related markers and the NLR reflect an active systemic inflammatory and stress response. This warrants enhanced screening for occult infections alongside measures such as optimized analgesia and temperature management to reduce the inflammatory burden. The observed biphasic pattern of lymphocyte and monocyte counts suggests immune activation followed by potential exhaustion. This attenuated and delayed response, particularly prominent in very old patients (≥ 80 years), likely reflects immunosenescence and identifies a clinically vulnerable phenotype. Consequently, enhanced infection surveillance, strict prevention, and reinforced supportive care are warranted for these high‐risk individuals. Therefore, this framework utilizes the dynamic changes in laboratory markers to assist clinicians in assessment, enabling earlier identification of higher‐risk patient profiles in terms of bleeding, inflammation, or immune status, which allows for more targeted preoperative optimization and risk mitigation.

### 4.4 Limitations

However, several limitations of this study should be noted. First, this study was retrospectively conducted in a single center; participants were those who had complete laboratory testing during the first 5 days post‐fracture before surgery, and patients operated on earlier were not represented. Therefore, these findings should not be interpreted as evidence to support delaying surgery, and their generalizability to other settings is unknown. Second, the sample size was relatively small (*n* = 60), which inevitably weakens the statistical power, especially in subgroup analyses. Third, this study focused on routinely available hematological and inflammatory‐immune markers but did not collect other more specific biomarkers or perioperative interventions, potentially resulting in residual confounding. Future prospective, multicenter, large‐sample studies are needed to validate these trajectories and to assess their clinical value.

## 5 Conclusions

This study descriptively depicts the dynamic changes in a series of hematologic and inflammatory‐immune markers within 1–5 days after hip fractures in elderly patients and suggested that several of them exhibit time‐dependent patterns that differ by fracture type and age group. These findings may help clinicians interpret routine laboratory tests in the early post‐fracture period and provide objective Supporting Information for perioperative assessment and optimization. However, future prospective studies are warranted to validate our findings and to assess their clinical value in risk stratification and patient outcomes.

## Author Contributions

All authors had full access to the data in the study and take responsibility for the integrity of the data and the accuracy of the data analysis. Conceptualization, Y.Z.Z., Y.B.Z. and X.T.L. Methodology, X.Q.C. and C.S.L. Investigation, S.S.Z. and Y.Q.L. Data curation, Y.N.H. and C.C.B. Writing – original Draft, Y.N.H. and C.C.B. Writing – review and editing, Y.N.H., Y.B.Z. and C.C.B. Supervision, Y.Z.Z., Y.B.Z. and X.T.L.

## Funding

This study was supported by the Hebei Provincial Department of Finance/Hebei Provincial Health Commission 2024 Government funded Clinical Medicine Excellent Talent Training Project (ZF2024075), and the Hygiene and Health Innovation Project of Major Project Assignment for Research and Development in Hebei Province (21377731D).

## Disclosure

All authors listed meet the authorship criteria according to the latest guidelines of the International Committee of Medical Journal Editors, and all authors are in agreement with the manuscript.

## Ethics Statement

This study was performed in line with the principles of the Declaration of Helsinki. Approval was granted by the Ethics Committee of the Third Hospital of Hebei Medical University (W2024‐014‐1).

## Consent

The research design adopted a retrospective approach and the data were fully anonymized, thus exempting the need for informed consent.

## Conflicts of Interest

The authors declare no conflicts of interest.

## Data Availability

The data that support the findings of this study are available from the corresponding author upon reasonable request.
